# Pharmacodynamic interactions between seletracetam and antiseizure comedications in the human photosensitivity model

**DOI:** 10.1111/epi.18420

**Published:** 2025-04-22

**Authors:** Wolfgang Löscher, Armel Stockis, Pavel Klein, Dorothee Kasteleijn‐Nolst Trenité

**Affiliations:** ^1^ Translational Neuropharmacology Lab, NIFE, Department of Experimental Otology of the Clinic of Otorhinolaryngology Hannover Medical School Hannover Germany; ^2^ PrevEp Bethesda Maryland USA; ^3^ UCB Pharma Clinical Pharmacology Braine‐l'Alleud Belgium; ^4^ Mid‐Atlantic Epilepsy and Sleep Center Bethesda Maryland USA; ^5^ Department of Neurosurgery and Epilepsy University Medical Center Utrecht Utrecht the Netherlands; ^6^ Nesmos Department, Faculty of Medicine and Psychology Sapienza University Rome Italy

**Keywords:** lamotrigine, levetiracetam, photoparoxysmal response, photosensitive epilepsy, synaptic vesicle glycoprotein 2A, valproate

## Abstract

We recently reported that seletracetam (SEL), a highly potent derivative of levetiracetam (LEV), reduces or abolishes the photoparoxysmal electroencephalographic response (PPR) to intermittent photic stimulation (IPS) in patients with epilepsy. Most of the 27 patients in this study were on comedication with different antiseizure medications (ASMs). Here, we reanalyzed the raw data of this clinical trial to determine which, if any, of the ASMs reduced (or increased) the effect of SEL on PPR in individual patients. This was possible because a group of six patients were not taking any ASM, and groups of similar size received different comedications. The effect size of SEL on the standard photosensitivity range (SPR) was calculated by the area under the effect curve from 0 to 8 h (AUEC [0–8]) as SPR change from predose. All patients experienced PPRs in response to IPS during placebo treatment, indicating that the PPR was resistant to treatment with their steady‐state ASMs. Oral single‐dose treatment with SEL reduced/abolished PPR in most (32/36) exposures, but significant effects of ASM comedication were found. Patients comedicated with LEV + lamotrigine or LEV + valproate exhibited significantly lower AUEC (0–8)s than patients without comedication, whereas no significant effects of lamotrigine or valproate alone were found. Despite the significant reduction of AUEC (0–8) in patients on comedication with LEV, SEL still reduced or abolished PPR in the majority (7/9) of exposures.

## 1 INTRODUCTION

Seletracetam (SEL) was discovered by a drug discovery program at UCB Pharma in which ~12 000 compounds were screened in vitro for binding affinity for synaptic vesicle glycoprotein 2A (SV2A), the main target of the benchmark antiseizure medication (ASM) levetiracetam (LEV); 1200 compounds were further screened in vivo for seizure protection in an animal model, the audiogenic seizure‐prone DBA/2 mouse model of reflex epilepsy.^1^ This led to the identification of two distinct antiseizure families with high affinity for SV2A. They were named after their lead compounds: SEL (a difluorovinyl derivative of LEV) and brivaracetam (BRV; a 4‐n‐propyl LEV homolog). Both SEL and BRV have a 1‐log‐unit higher affinity for SV2A than LEV and are more selective.^1^, ^2^, ^3^ Both racetams are also much more lipophilic and markedly more potent than LEV in acute and chronic animal models of focal and generalized seizures.^4^ Both SEL and BRV underwent phase I and IIa clinical studies, but UCB Pharma decided to evaluate only BRV in large multicenter phase III trials, leading to US Food and Drug Administration approval of BRV in 2015 for the treatment of focal onset seizures.^5^ At least in part, the decision to choose BRV and not SEL was based on the broader spectrum of antiseizure activities of BRV in animal models.^4^ SEL was progressed by UCB as a BRV backup should the latter fail, which did not occur.

Based on the much higher antiseizure potency of SEL versus LEV and BRV,^4^ SEL is currently in development by PrevEp as the first intranasal nonbenzodiazepine (non‐BDZ) seizure rescue therapy. For this indication, it is important to know whether SEL is effective in patients who do not respond to chronic treatment with LEV or BRV. We have recently reported the outcome of a phase II randomized, placebo‐controlled, single‐blind trial with SEL in photosensitive epilepsy patients.^6^ In this proof‐of‐principle trial in the “photosensitivity model,” the effect of acute oral doses of SEL (.5, 1, 2, 4, 10, and 20 mg) on the photoparoxysmal electroencephalographic response (PPR) to intermittent photic stimulation (IPS) was evaluated in a group of 27 patients with a total of 36 SEL exposures. The majority of these patients were on chronic treatment with ASMs, including LEV, which seemed to reduce the effect of SEL, but potential interactions were not examined in detail.

The purpose of the present study was to determine which, if any, of the ASMs reduced (or increased) the effect of SEL on PPR in individual patients. This analysis was possible because a relatively large group of patients did not receive any ASM comedication together with SEL, and groups of similar size received different comedications. In previous clinical trials in photosensitive patients with LEV^7^ and BRV,^8^ drug–drug interaction analyses were not possible because the study groups were too small and only four of 12 patients in the LEV trial and three of 18 patients in the BRV trial did not receive ASM comedication. This is the first (and only) clinical study in epilepsy patients that examines the interaction between SEL and ASMs such as LEV.

## 2 MATERIALS AND METHODS

For this study, the raw data of the phase IIa trial with SEL in 27 photosensitive epilepsy patients^6^ were reanalyzed. All details on patients and trial characteristics were reported in our previous study and are not repeated here. The majority of the patients had spontaneous seizures at the time of the trial. In each patient, the standard photosensitivity range (SPR) was derived from upper and lower sensitivity thresholds as measured with a standard amount of flash frequencies starting at 2 Hz and going upward until a generalized PPR was seen and then from 60 Hz flashing going downward. On the first placebo day (day −1), photic SPR determinations were done at hours .5, 1, 2, 4, 6, and 8 after dosing, and on the second day (day 1), single oral doses of SEL (.5, 1, 2, 4, 10, or 20 mg) were given with IPS testing at the same hours postdosing. As reported,^6^ placebo administration did not affect PPR in most patients.

The following readouts were used for the reanalysis of drug–drug interactions in the eye closure condition:

1. Response to SEL in terms of photosensitivity was classified as “no change,” “response but no abolishment,” and “complete abolishment.” “Complete abolishment” meant that at all frequencies, there was no PPR at least at one time point. In practice, all full responders reacted for at least 8 h with complete suppression. Partial response (i.e., “response but no abolishment”) was a reduction in PPR by at least three steps and lasted in general for hours.
2. The area under the effect curve from 0 to 8 h (AUEC [0–8]) was calculated as SPR change from predose versus time, using the linear trapezoidal rule. The AUEC (0–8) is a quantitative measure of effect size that allows determining whether comedication with an ASM affected the efficacy of SEL to reduce PPR.

Plasma levels of SEL and ASMs were determined in parallel to the SPR determinations, and we previously found no robust evidence for any pharmacokinetic drug–drug interactions.^6^ Thus, plasma levels are not reported again here.

The significance of differences between responses to SEL in the different comedication groups was analyzed by Barnard test. Kruskal–Wallis analysis of variance for nonparametric data followed by Dunn multiple comparisons tests was used for analysis of group differences in AUEC (0–8)s. As described above and shown in Table 1, there were 27 patients but 36 exposures, because some individuals were tested at more than one dose, which could bias the results.

**TABLE 1:** Effects of comedication with antiseizure medications on seletracetam's effect on the SPR (eye closure condition) on day 1.

Patient #	Sex/age, years	Spontaneous seizures	Comedication	Dose of comedication, mg/day	SEL dose, mg	Effect of SEL on day 1 (change from predose)			AUEC (0–8)
						Complete	Partial	None	
No comedication, *n* = 7									
18	F/21	Yes	–	–	2	+			−53.75
12	F/20	Yes	–	–	2	+			−62.0
20	F/20	Yes	–	–	2	+			−51.75
11	F/22	No	–	–	10		+		−60.0
12	F/20	Yes	–	–	10	+			−62.0
3	F/51	No	–	–	10	+			−62.0
24	F/24	Yes	–	–	20	+			−7.75
Group response to SEL						6/7	1/7	0/7	
LEV + LTG, *n* = 7									
10	F/36	Yes	LEV + LTG	1000 + 200	2			+	−5.0
9	F/19	Yes	LEV + LTG	2000 + 325	2		+		−22.0
14	F/39	Yes	LEV + LTG	1000 + 200	4			+	2
10	F/36	Yes	LEV + LTG	1000 + 200	10	+			−17.5
14	F/39	Yes	LEV + LTG	1000 + 200	20	+			−35.3
22	F/34	Yes	LEV + LTG	500 + 100	20		+		−5.0
9	F/19	Yes	LEV + LTG	2000 + 325	20		+		−27.75
Group response to SEL						2/7^a^	3/7	2/7	
LTG alone, *n* = 4									
6	F/19	ND	LTG	400	1		+		−35.5
1	F/25	Yes	LTG	200	10	+			−15.5
2	F/20	Yes	LTG	300	10	+			−51.25
6	F/19	ND	LTG	400	10		+		−23.25
Group response to SEL						2/4	2/4	0/4	
LEV + VPA, *n* = 2									
17	M/27	ND	LEV + VPA	1000 + 1000	4	+			−4.5
26	F/20	Yes	LEV + VPA	1000 + 750	20		+		−13.0
All LEV, *n* = 9									
Group response to SEL						3/9	4/9	2/9	
VPA alone, *n* = 11									
15	F/23	Yes	VPA	1000	.5	+			−7.0
23	F/20	Yes	VPA	800	.5	+			−52.75
27	M/17	Yes	VPA	875	.5		+		−12.0
27	M/17	Yes	VPA	875	20		+		−19.25
28	M/17	ND	VPA	875	.5			+	−3.0
25	F/23	Yes	VPA	500	1	+			−6.25
8	M/18	ND	VPA	1000	2	+			−77.5
16	F/25	Yes	VPA	1000	2			+	−5.75
13	F/18	Yes	VPA	500	4		+		−33.0
25	F/23	Yes	VPA	500	20	+			−62.0
28	M/17	ND	VPA	875	20		+		−30.75
Group response to SEL						5/11	4/11	2/11	
Other ASMs, *n* = 5									
4	F/37	Yes	CBZ	400	1		+		−26.75
19	F/26	ND	TPM	50	4	+			−76.0
21	F/35	Yes	LTG + TPM	200 + 200	.5		+		−39.5
7	F/25	Yes	PB + PHT	65 + 50	1	+			−56.0
7	F/25	Yes	PB + PHT	65 + 50	4	+			−50.75
Group response to SEL						3/5	2/5	0/5	
Overall response to SEL						19/36	13/36	4/36	

*Note*: Pharmacodynamic outcomes (photoparoxysmal EEG response and SPR) were analyzed by an independent central EEG reader. Overall, SEL was tested in 36 exposures in 27 patients. Details of patients other than those shown here are given in Table 1 of Kasteleijn‐Nolst Trenité et al.^6^ (for cross‐reference, the patient number used in Table 1 of this previous paper is also used here). AUEC (0–8) represents the change in SPR from predose (for the eye closure condition) and represents a quantitative measure of the effect size of SEL.

Abbreviations: AUEC (0–8), area under the effect curve from 0 to 8 h; CBZ, carbamazepine; EEG, electroencephalographic; F, female; LEV, levetiracetam; LTG, lamotrigine; M, male; ND, not determined; PB, phenobarbital; PHT, phenytoin; SEL, seletracetam; SPR, standard photosensitivity range; TPM, topiramate; VPA, valproate.

^a^ Significant difference in the frequency of responses to SEL without comedication (*p* = .04273).

## 3 RESULTS

**FIGURE 1:**
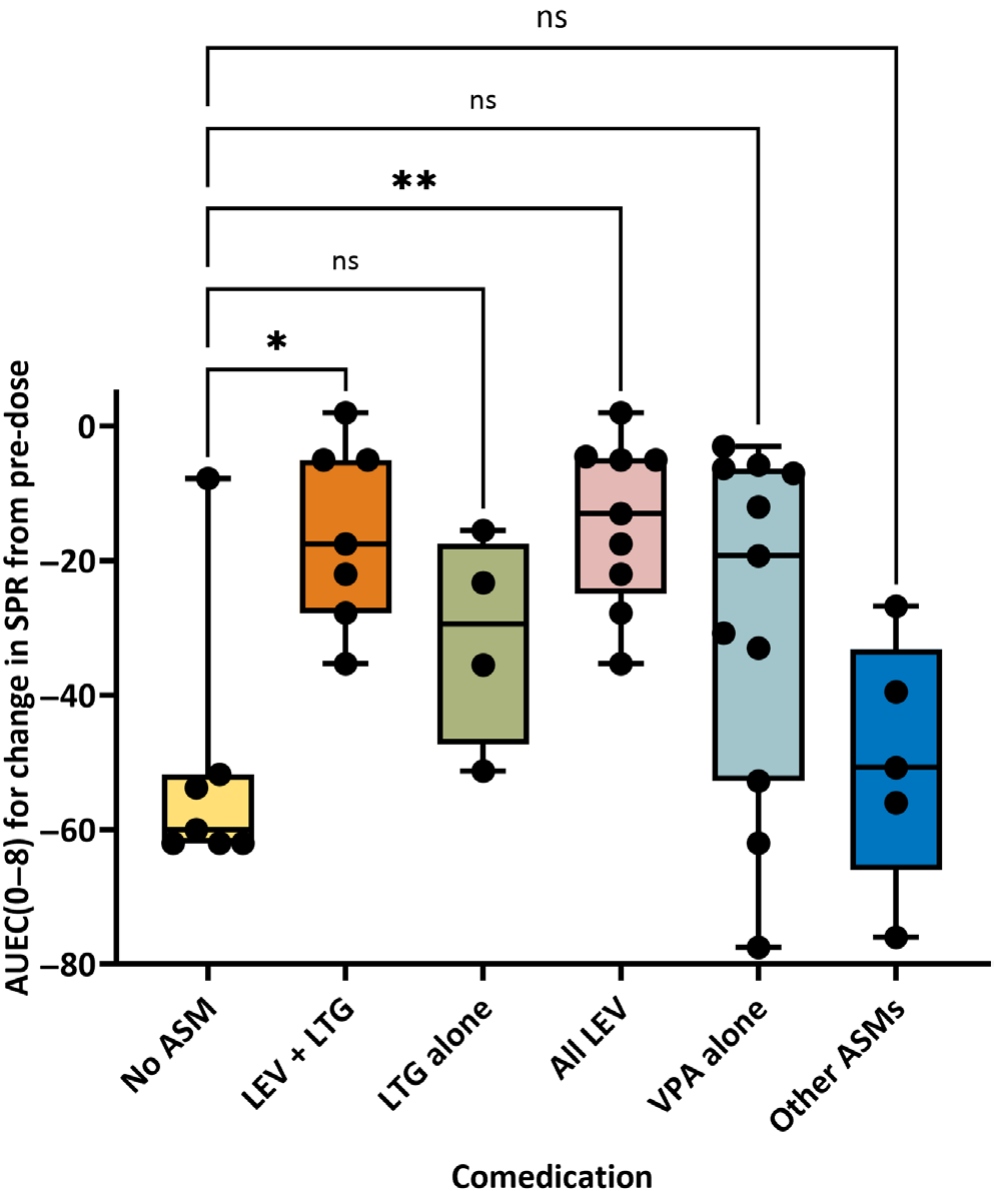
Effect of comedication with antiseizure medications (ASMs) on the efficacy of seletracetam (SEL) to reduce the standard photosensitivity range (SPR) after intermittent photic stimulation (IPS) in 27 patients with photosensitive epilepsy. The efficacy to reduce SPR after IPS is shown by the area under the effect curve from 0 to 8 h (AUEC [0–8]), calculated as the change from predose in SPR for the eye closure condition. SEL was tested following oral administration of single doses, ranging from .5 to 20 mg. Because the same dose range was tested in all subgroups (see Table 1), all values are shown together for each group. Data are illustrated as box plots with whiskers from minimum to maximal values; the horizontal line in the boxes represents the median value. In addition, individual data are shown. Statistical analysis by Kruskal–Wallis analysis of variance indicated significant differences across comedication groups (*p* = .0058). Dunn multiple comparisons tests indicated a significant difference between the group without comedication (“No ASM”) and the group comedicated with levetiracetam (LEV) and lamotrigine (LTG) alone, or combined with two patients on LEV plus valproate (VPA; “All LEV”); significance is indicated by asterisks (*p = .0215, **p = .0064). ns, not significant.

Table 1 illustrates the responses to different doses of SEL in the 36 individual exposures in 27 patients with photosensitive epilepsy. In seven of 36 exposures, there was no ASM comedication; in another seven exposures, patients were chronically treated with LEV and lamotrigine (LTG); two other patients received LEV and valproate (VPA); in 11 of 36 exposures, patients received VPA monotherapy; in five of 36 exposures, patients were treated with other ASMs (Table 1). No patients were treated with LEV monotherapy. Overall, an effect of SEL was determined in 32 of 36 exposures (complete abolishment of PPR in 19/36 and partial suppression in 13/36 exposures). Individual data and subgroup analyses are illustrated in Table 1 and Figure 1.

In six of the seven exposures without comedication, SEL abolished PPR at doses ranging from 2 to 20 mg; partial response was seen in one exposure. In another group of seven exposures, in which patients received comedication with LEV and LTG, SEL abolished PPR in only two of seven exposures, which was statistically significant from the first group (*p* = .04273). Overall, in five of seven exposures, patients in the LEV/LTG group responded to SEL. When the median AUEC (0–8) values of the two groups were compared, AUEC (0–8)s were significantly lower (*p* = .0215) in the LEV/LTG group (Figure 1), indicating a drug–drug interaction. A third group of patients received comedication with LTG alone, which did not affect the response to SEL (Table 1, Figure 1), indicating that the significant reduction of SEL's effect on AUEC (0–8) by comedication with LEV and LTG was due to LEV.

Two patients were on comedication with LEV and VPA. When these two patients were added to the LEV + LTG group, three of nine patients responded to SEL with PPR abolishment, which was not different from the SEL group without comedication (*p* = .5688). Overall, six of nine patients responded to SEL in the LEV group (Table 1). AUEC (0–8) values in this group were significantly (*p* = .0064) lower than these values in the SEL group without comedication (Figure 1). Daily doses of LEV ranged between 500 and 2000 mg (median = 1000 mg), but there was no obvious relationship between the dose of LEV and the attenuated response to SEL (Table 1).

In 11 exposures, patients were on comedication with VPA. In the 11 SEL exposures in this group, no effect was seen in two exposures, but the overall response to SEL in this group was not significantly different from the group without comedication (Table 1, Figure 1). The group on VPA comedication was the only one in which complete suppression of PPR was determined at .5 mg SEL (seen in two patients). Several patients received comedication with carbamazepine, topiramate (TPM), or phenobarbital (PB) and phenytoin, which did not result in any obvious reduction of the effect of SEL on PPR.

When analyzing all 36 SEL exposures, there was a significant relationship between dose and effect of SEL (see Kasteleijn‐Nolst Trenité et al.^6^). However, such a relationship was less obvious in the subgroups analyzed here (see Table 1).

## 4 DISCUSSION

All patients enrolled in the present study experienced PPRs in response to IPS during placebo treatment, although the majority of the patients were on chronic treatment with ASMs (LEV, VPA, LTG) known to suppress PPR in photosensitive epilepsy patients.^9^, ^10^ This may indicate that the PPR in these patients was resistant to treatment with steady‐state doses of ASMs and that this resistance could be overcome by add‐on treatment with SEL in most patients. Importantly, this was also true for patients on comedication with LEV. In seven of nine SEL exposures with chronic LEV, a response to SEL was obtained compared to seven of seven exposures without any ASM comedication. Although complete suppression of PPR by SEL tended to be lower in the LEV group (3/9 vs. 6/7), this difference was not statistically significant. However, analysis of AUEC (0–8) indicated a significantly reduced effect size of SEL in the LEV group.

Both SEL and LEV act as modulators of SV2A, which is considered the main mechanism of action of these racetams.^4^ Thus, one would expect that comedication with LEV (and resistance of PPR to LEV) would abolish the effect of SEL. This was not the case. It has been shown previously that LEV, BRV, and SEL bind to the SV2A protein at closely related sites but interact with these sites differently.^4^, ^11^, ^12^, ^13^ Data for LEV and BRV suggest that these drugs recognize, induce, or stabilize different conformations of the SV2A protein, which may provide the molecular rationale for their distinct pharmacodynamic properties.^4^ This also explains why epilepsy patients who are resistant to LEV may benefit from switching to BRV.^5^, ^14^, ^15^ SEL not only differs from LEV in its much higher affinity for SV2A,^16^ but it also dissociates much more slowly from SV2A than LEV (Michel Gillard, unpublished data). This, together with the higher affinity, may explain why SEL is much more potent than LEV both preclinically and clinically.^4^, ^6^ However, all three racetams also affect other targets that may contribute to their antiseizure effects.^4^ Importantly, SEL has been shown to inhibit high‐voltage‐activated Ca^2+^ currents (HVACCs) 50 times more potently than LEV.^17^ The half‐maximal inhibitory concentration (IC_50_; .27 μmol·L^−1^) of SEL for inhibiting HVACCs is close to its IC_50_ (.14 μmol·L^−1^) at SV2A.^18^ In vitro experiments with toxins that target different subtypes of HVACCs suggested that N‐type Ca^2+^ channels, and partly Q‐type subtypes, are preferentially targeted by SEL.^17^ Other ASMs that inhibit HVACCs at therapeutically relevant drug levels include LTG, TPM, and PB but not VPA.^19^ However, the combination of effects on both SV2A and HVACCs is unique for SEL and may explain why SEL inhibited PPR despite the presence of LEV. This finding may suggest that in clinical practice the two drugs could be combined without significantly reducing the benefits of SEL.

Whereas comedication with LTG did not reduce SEL's effect on PPR, patients on comedication with VPA tended to exhibit lower responses to SEL, although the difference to SEL alone was not statistically significant. In a previous study with LEV in 12 photosensitive patients, the majority of patients were on comedication with VPA and only four patients were without comedication, so a meaningful subgroup analysis was not possible.^7^ Similarly, in the previous study with BRV in 18 photosensitive patients, only three patients were not comedicated, nine patients were comedicated with VPA, and only one patient was on comedication with LEV, so drug–drug interaction analyses were not possible.^8^

It is worth noting that SEL is the only non‐BDZ ever tested in the photosensitivity model that exerts effects on PPR at doses as low as effective doses of BDZs in this model.^9^, ^10^ In addition to its remarkably potency, SEL combines high lipophilicity with high water solubility, which makes it an interesting candidate for intranasal treatment of acute repetitive seizures.

In conclusion, as reported previously,^6^ SEL is very potent and effective in reducing or abolishing PPR in patients with epilepsy. Comedication with LEV reduces but does not abolish the anti‐PPR effect of SEL, whereas several other ASMs showed no obvious drug–drug interaction with SEL. The promising efficacy of SEL in the photosensitivity model has been substantiated by the preliminary outcome of two multicenter phase IIa trials with SEL add‐on treatment in patients with drug‐resistant focal onset seizures (NCT00152503 and NCT00152451). Overall, the available preclinical and clinical data indicate that SEL is a promising ASM candidate, one with a potent, broad spectrum of seizure protection and a high central nervous system tolerability in animal models and high potency, straightforward pharmacokinetics, and good tolerability in epilepsy patients.

## AUTHOR CONTRIBUTIONS

Wolfgang Löscher reanalyzed the raw data and was involved in the interpretation of the data and the conceptualization and writing of the original draft of the manuscript. Dorothee Kasteleijn‐Nolst Trenité was involved in the clinical study design and execution and the editing of the manuscript. Armel Stockis was involved in the analysis and interpretation of the data and editing of the manuscript. Pavel Klein was involved in the interpretation of the data and editing of the manuscript. All authors reviewed and approved the manuscript.

## FUNDING INFORMATION

The clinical seletracetam study was fully sponsored by UCB (Brussels, Belgium). Publishing and analyzing the data are a private enterprise by the authors with the consent of UCB. UCB did not participate in the generation or revision of the manuscript.

## CONFLICT OF INTEREST STATEMENT

W.L. is cofounder and CSO of PrevEp (Bethesda, MD, USA). He has received consultancy fees in the past 5 years from Lundbeck, Angelini, Clexio, Selene, Axonis, SynapCell, Sintetica, ND Capital, Atlas Venture, Cogent Biosolutions, Ovid, Idorsia, and Addex. D.K.N.T. has received consultancy fees in the past 5 years from UCB, Otsuka, SK, Jazz, and Praxis. A.S. is a former employee of UCB Pharma and has received consultancy fees in the past 5 years from UCB, Roche, and EyeD Pharma. P.K. has served as a consultant, advisory board member, or speaker (2020–2024) for Abbott, Angelini, Aquestive, Arvelle Therapeutics, Aucta Pharmaceuticals, Dr. Reddy's, Eisai, GRIN Therapeutics, Neurelis, Neurona, Paladin, SK Life Science, Sunovion, UCB Pharma, UNEEG, and UniQure; is a member of the medical advisory board of Alliance and of the scientific advisory boards of OB Pharma and NEUmiRNA; is the CEO of PrevEp; and has received research support from CURE/Department of Defense and the NIH/SBIR. We confirm that we have read the Journal's position on issues involved in ethical publication and affirm that this report is consistent with those guidelines.

## Data Availability

The data that support the findings of this study are available from the corresponding author upon reasonable request.
